# 磁性三维氮掺杂碳纳米材料对6种双酚类化合物的吸附性能及其在泡腾分散微萃取中的应用

**DOI:** 10.3724/SP.J.1123.2022.03041

**Published:** 2022-12-08

**Authors:** Tingting LIU, Qi WANG, Hanzhang YE, Jia KONG, Yuhao LI, Jingjing GU, Yongli DING, Zhan’en ZHANG, Xuedong WANG

**Affiliations:** 1.苏州科技大学环境科学与工程学院, 江苏 苏州 215009; 1. School of Environmental Science and Engineering, Suzhou University of Science and Technology, Suzhou 215009, China; 2.江苏省环境科学与工程重点实验室, 江苏 苏州 215009; 2. Jiangsu Key Laboratory of Environmental Science and Engineering, Suzhou 215009, China

**Keywords:** 磁性碳复合材料, 泡腾分散固相微萃取, 高效液相色谱-荧光检测, 双酚类, 吸附剂, 吸附机理, magnetic carbon-based nanocomposite, effervescent reaction-assisted dispersive solid-phase microextraction, high performance liquid chromatography-fluorescence detection (HPLC-FLD), bisphenol, adsorbent, adsorption mechanism

## Abstract

采用简单高温煅烧法成功制备了磁性钴镍基氮掺杂三维碳纳米管与石墨烯复合材料(CoNi@NGC),将其作为吸附剂用于水体中6种双酚类化合物(BPs)的吸附性能和机理研究。将CoNi@NGC复合纳米材料用作萃取介质,运用酸碱泡腾片的CO_2_强力分散作用,开发了泡腾反应强化的分散固相微萃取前处理方法,结合高效液相色谱-荧光检测(HPLC-FLD)快速定量饮料中痕量BPs。采用扫描电镜、透射电镜、傅里叶红外光谱、氮气吸脱附、X射线光电子能谱和磁滞回线等技术手段对材料形貌结构进行表征,结果显示:该吸附剂成功实现氮元素的掺杂,且具有较大的比表面积(109.42 m^2^/g)、丰富的孔径及较强的磁性(17.98 emu/g)。吸附剂投加量、pH、温度、时间等因子优化试验表明:当pH=7,在初始质量浓度为5 mg/L的BPs混合溶液中投加5 mg CoNi@NGC, 298 K反应5 min,对双酚M(BPM)、双酚A(BPA)的吸附率分别高达99.01%和98.21%。作用90 min时对双酚Z(BPZ)、BPA、BPM的吸附率近100%。在吸附过程中,BPs与CoNi@NGC之间的整个吸附过程主要受氢键、静电作用和*π-π*共轭作用共同控制。整个吸附过程符合Freundlich吸附等温线模型和准二级动力学方程,吸附自发进行。进一步将CoNi@NGC作为萃取介质制备成磁性泡腾片,利用泡腾分散微萃取技术高效富集和提取6种盒装饮料中的BPs,优化了影响富集效果的泡腾片的存在与否、洗脱剂种类、洗脱时间、洗脱体积等关键因子,在最佳萃取条件下(pH=7,投加5 mg CoNi@NGC, 2 mL丙酮洗脱6 min),结合HPLC-FLD,新开发的泡腾分散微萃取方法提供的检出限为0.06~0.20 μg/L,定量限为0.20~0.66 μg/L,日内和日间精密度分别为1.44%~4.76%和1.69%~5.36%,在实际样品中不同水平下的加标回收率为82.4%~103.7%,在桃汁中检测到BPA和双酚B(BPB)分别为2.09 μg/L和1.37 μg/L。再生试验表明该吸附材料至少可以重复使用5次以上,显著降低了分析的试验成本。与其他方法相比,该方法具有灵敏度高、萃取速度快、环境友好等优点,在常规食品污染监测中具有较强的应用价值。

## Abstract (en)

Herein, we successfully prepared magnetic Co/Ni-based N-doped 3D carbon nanotubes and graphene nanocomposites (CoNi@NGC) using a simple high-temperature calcination method. The CoNi@NGC nanocomposites were used as adsorbents to study their adsorption performances and underlying kinetic mechanisms for six types of bisphenol compounds (BPs) in water. They were also used as extractants, and acid-base effervescent tablets were used to enhance extractant dispersion with the aid of vigorous CO_2_ bubbling. Thus, a novel pretreatment method was developed, denoted effervescent reaction-assisted dispersive solid-phase microextraction (ER-DSM), which was combined with high performance liquid chromatography-fluorescence detection (HPLC-FLD) to rapidly quantify trace-level BPs in several drinks. The morphology and structure of the CoNi@NGC adsorbent were characterized in detail using scanning electron microscopy (SEM), transmission electron microscopy (TEM), Fourier transform infrared spectroscopy (FT-IR), N_2_ adsorption and desorption (BET-BJH), X-ray photoelectron spectroscopy (XPS), and vibrating sample magnetometry (VSM). The CoNi@NGC nanocomposites were successfully doped with N and exhibited large specific surface areas (109.42 m^2^/g), abundant pores, and strong magnetic properties (17.98 emu/g).

Key parameters were rigorously optimized to maximize the adsorption performance of CoNi@NGC, including adsorbent dosage, solution pH, temperature, and time. Under the constant conditions of pH=7, 5 mg of CoNi@NGC, initial BP concentrations of 5 mg/L, and 5 min of shaking at 298 K, the adsorption percentages of bisphenol M (BPM) and bisphenol A (BPA) reached respective maxima of 99.01% and 98.21%. Remarkably, those of bisphenol Z (BPZ), BPA, and BPM reached almost 100% after 90 min. The adsorption between the BPs and CoNi@NGC was mainly governed by hydrogen bonds, electrostatic interactions, and *π-π* conjugation. The entire adsorption process was consistent with Freundlich adsorption and a quasi-second-order kinetic equation, representing spontaneous adsorption. Via integration with HPLC-FLD, ER-DSM was used to rapidly extract and analyze trace-level BPs in six types of boxed drinks. Critical factors were optimized individually, including the type of eluent and elution time and volume, which influenced the enrichment effect. Under the optimized extraction conditions (pH=7, 5 mg CoNi@NGC, elution with 2 mL acetone for 6 min), the limits of detection and quantification of the novel extraction method were 0.06-0.20 and 0.20-0.66 μg/L, respectively. The intra- and inter-day precisions spanned the ranges 1.44%-4.76% and 1.69%-5.36%, respectively, and the recoveries in the actual samples were in the range 82.4%-103.7%. Moreover, the respective residual levels of BPA and BPB in peach juice samples were 2.09 and 1.37 μg/L. Regeneration studies revealed that the CoNi@NGC adsorbent could be reused at least five times, which significantly reduced the cost of evaluation. In summary, compared to other methods, this method displays the advantages of a high sensitivity, rapid extraction, and environmental friendliness, thereby exhibiting considerable potential for use in conventional monitoring of trace-level BPs in food matrices.

双酚类(BPs)污染物作为具有雌激素活性的内分泌干扰物,因其酚环由碳桥键或其他官能团连接而成,在环境样本中具有较强的特异性和较长的半衰期,易迁移富集且难以代谢^[1]^。双酚A(BPA)及其类似物是生产聚碳酸塑料和环氧树脂中不可或缺的添加剂,近年来在水体、食品、废旧电子产品等样本中均被检测出^[2]^。它们可通过食物链迁移至人体内富集,由于其具有亲脂性和持久性,在生物体内可残留数十年甚至更长时间,可引起生殖系统和神经系统的病变。选择优异的方法去除或检测环境介质中的双酚类污染物,对生态环境和人体健康均有重要意义^[3]^。

目前,去除环境水体中BPs的主要方法有催化降解法^[4]^、生物法^[5]^、吸附法^[6]^等。其中,吸附法被公认是一种经济高效、简单易行的去除有机污染物的方法。选择具有制备方法简单、环境友好、价格低廉、吸附效率高、重复性能好、易于快速分离等优点的吸附剂是实现BPs去除的重要环节^[7]^。碳材料基吸附剂性质稳定、价格低廉且对环境友好,活性炭、碳纤维、多孔碳及各类纳米碳材料均已被证明对抗生素、内分泌干扰物等具有良好的吸附效果^[8]^。纯碳材料的性质单一,应用范围有限,对碳材料进行改性或与其他材料复合可以弥补其缺陷,通过不同材料间的协同作用可进一步提升其作为吸附剂的实用性^[9⇓-11]^。Lv等^[12]^制备了一种孔径丰富、晶体结构稳定、热稳定性良好、官能团丰富的*β*-环糊精修饰的多孔纤维素纳米纤维膜(CA-P-CDP),大大提高了水中微量双酚A、双酚S(BPS)和双酚F(BPF)污染物的吸附效率。在25 ℃、pH=7、投加量为0.1 g/L条件下吸附15 min,三者的吸附量分别为50.37、48.52和47.25 mg/g。Shao等^[13]^用废弃烟蒂制备出了一种吸附容量高(865 mg/g)、反应速度快(186.9 mg/(g·min))、重复使用性能优异的多孔炭(AC-800)材料,通过对其吸附机理探究发现,远距离疏水作用和短距离分散力的协同作用对BPA在AC-800上的吸附有重要贡献。Marrakchi等^[14]^以碱处理海藻为原料,用H_3_PO_4_浸渍热解,利用C_3_H_6_N_6_改性制备了氮掺杂介孔活性热解碳作为吸附剂应用于吸附水中的双酚A发现,材料表面成功引入含氮官能团使得对双酚A的去除率由59.22%提高至91.48%。多孔结构的构筑及氮原子的成功掺杂对提高BPs的效率有着重要作用。

近年来,BPs在环境介质、玩具、食品等被检测出且呈增多趋势,对BPs的高通量检测也成为研究的热点^[2,15]^。常用的同时实现多种双酚类物质的分析方法有超高效液相色谱-串联质谱法^[16]^、气相色谱-质谱联用法(GC-MS)^[17]^等,此类方法灵敏度高,检测种类多,但所需仪器配置高,操作复杂。若采用优异的前处理方法,对各类介质中的BPs进行富集浓缩,则可使用较为简便的高效液相色谱-荧光检测法(HPLC-FLD)检测多种BPs。微萃取技术的出现克服了传统液液萃取法和固相萃取法消耗有机试剂多的问题,其中固相微萃取法中,选择优异的吸附剂,可大大提高富集效果^[2,18,19]^。在固相微萃取法中,增加吸附剂的磁性可实现萃取过程中吸附剂的快速分离;增加由酸源、碱源反应产生CO_2_气泡的泡腾片剂技术,有助于吸附剂的快速分散,实现快速萃取^[20,21]^。

基于此,本实验选择以六水合氯化钴(CoCl_2_·6H_2_O)、四水合醋酸镍Ni(CH_3_COO)_2_·4H_2_O、三聚氰胺(C_3_H_6_N_6_)和氧化石墨烯(GO)为原料,在体系中添加十六烷基三甲基溴化铵(CTAB)制备了在石墨烯片层原位生长碳纳米管且同时实现钴镍合金和氮元素共修饰的三维复合材料(CoNi@NGC)。采用扫描电子显微镜(SEM)、透射电子显微镜(TEM)、傅里叶红外变换光谱(FT-IR)、N_2_吸脱附等温线测试(BET-BJH)、X射线光电子能谱(XPS)和磁滞回线测试(VSM)等技术对材料进行表征,并将其用作吸附剂,优化吸附条件,结合HPLC-FLD同时吸附水体中6种BPs的吸附效果,并探究其吸附机理。将该吸附剂与泡腾片剂相结合,建立了泡腾反应强化的磁性分散固相微萃取技术(MSPE)与HPLC-FLD结合使用的新方法,用以检测果汁中6种BPs。

## 1 实验部分

### 1.1 试剂与仪器

BPA(C_15_H_16_O_2_)购自日本WAKO公司;双酚Z(BPZ, C_20_H_18_O_2_)、BPF(C_13_H_12_O_2_)、双酚M(BPM, C_13_H_12_O_2_)、CTAB购于美国Sigma-Aldrich公司;双酚B(BPB, C_16_H_18_O_2_),双酚AP(BPAP, C_20_H_18_O_2_)购自美国Accustandard公司;所有试剂纯度均大于99%,且未再做任何纯化处理。C_3_H_6_N_6_、CoCl_2_·6H_2_O和Ni(CH_3_COO)_2_·4H_2_O购于瑞士Adamas公司;GO购于南京先丰纳米材料科技有限公司;色谱级甲醇(CH_3_OH)、乙腈(CH_3_CN)购于美国Tedia公司。L(+)-酒石酸(TTA, AR)和无水碳酸钠(Na_2_CO_3_, AR)购于国药化学试剂上海有限公司,色谱级丙酮购于美国Tedia公司。以甲醇配制上述BPs的母液(1 mg/mL)于-4 ℃暗处保存,使用前用甲醇逐级稀释至所需工作浓度。

场发射扫描电子显微镜(Quanta250;美国FEI),能谱仪(EDS)(Inca X-act;英国 Oxford),透射电子显微镜(TecnaiG2F30;美国FEI),傅里叶变换红外光谱仪(NicoletiS5;美国ThermoFisher), X射线光电子能谱仪(K-Alpha+型;美国ThermoFisher), X射线衍射仪(D8 Advance;德国Bruker),磁滞回线测试仪(MPMSXL-7;美国Quantum Design),比表面积及孔径分析仪(ASAP 2460;美国Micro),岛津高效液相色谱仪(LC-20AT型,配有SIL-20A自动进样盘和FLD、RF-20A荧光检测器;日本Shimadzu)。

### 1.2 高效液相色谱条件

色谱柱:岛津Shim-pack GIST C18色谱柱(250 mm×4.6 mm, 5 μm);流动相:乙腈-水,0~9 min体积比为50∶50, 9~20 min体积比为70∶30;流速:1.0 mL/min;柱温30 ℃;进样体积:10 μL;荧光检测器,激发和发射波长分别为233 nm和303 nm。

### 1.3 CoNi@NGC纳米复合材料的制备

称取10 mg GO超声分散于20 mL超纯水中形成溶液A;称取0.5 g CoCl_2_·6H_2_O和0.5 g Ni(CH_3_COO)_2_·4H_2_O超声溶解于10 mL超纯水中形成溶液B;称取0.5 g CTAB和5 g C_3_H_6_N_6_超声溶解于40 mL超纯水中形成溶液C;在搅拌条件下,将溶液B和溶液C依次逐滴滴加到溶液A中,升温至80 ℃继续搅拌3 h后将浓缩后的混合液进行冷冻干燥得到淡紫色固体。将该淡紫色前驱体置于氮气气氛管式炉中,进行程序升温煅烧,首先以2.5 ℃/min升温至500 ℃恒温1 h,随后继续以同样的升温速率升温至900 ℃煅烧1 h,可得到黑色粉末状固体;最后将该黑色粉末用200 mL 1 mol/L H_2_SO_4_搅拌酸洗12 h后离心,用超纯水和无水乙醇交替洗涤数次后于60 ℃真空烘箱中烘干备用。

### 1.4 CoNi@NGC纳米复合材料吸附BPs的实验

于250 mL锥形瓶中配制一系列100 mL含6种BPs(5 mg/L)的混合溶液,均加入5 mg CoNi@NGC纳米复合材料吸附剂,将所有锥形瓶放置在恒温振荡箱中放置25 ℃,振荡速度为200 r/min,于3、5、10、30、60、90、120 min时各取1 mL溶液,用磁铁将吸附剂与样品溶液分离,用HPLC测定上清液中BPs浓度。吸附量(*Q*, mg/g)和去除率(*η*)的计算公式如下:


(1) *Q=*(*C*_0_*-C*_t_)*V/W*

(2) *η*=(*C*_0_*-C*_t_)/*C*_0_×100%

其中:*C*_0_为初始BPs的质量浓度,mg/L; *C*_t_为吸附后BPs的质量浓度,mg/L; *V*为所取溶液量,mL; *W*为吸附剂质量,g。

### 1.5 实际样品前处理

从苏州当地某超市购买了6种盒装饮料:桃汁、冰糖雪梨汁、酸梅汁、蜂蜜柚子茶、橙汁、草莓乳酸菌。首先,量取5 mL样品和5 mL超纯水于50 mL离心管中振荡稀释1 min后于4 ℃冰箱中保存15 min,随后以5000 r/min速率离心10 min,用0.45 μm滤膜过滤上清液,所得溶液可在4 ℃冰箱中保存,并于一周内使用。

### 1.6 泡腾辅助磁性固相微萃取实验

将5 mg CoNi@NGC、150 mg TTA和106 mg无水Na_2_CO_3_研磨均匀,使用5T/8MM压片机压制磁性泡腾片。取5 mL预处理后的样品于15 mL离心管中,加入磁性泡腾片,在管中自下而上生成大量小气泡,使CoNi@NGC材料能够均匀快速分散。随后在离心管的底部放置外部磁铁来收集富含分析物的CoNi@NGC吸附剂,除去上清液,沉淀用2 mL丙酮洗脱6 min,收集洗脱液,在N_2_气流下氮吹至干,然后将其重溶于400 μL甲醇中,取10 μL过滤后的溶液进样分析。

## 2 结果与讨论

### 2.1 材料的形貌结构分析

采用SEM、TEM和EDS对CoNi@NGC形貌成分进行分析,如图1a,b所示,片层材料表面生长有管状材料,两种形貌碳材料的复合成功构建出蓬松的三维复合结构。通过TEM(图1c)表征可以看出,该材料呈现出片层与管状相结合,且这些材料中包裹有纳米颗粒。通过高分辨TEM(图1d)结果可以看出外层材料晶格间距为0.34 nm,与石墨化碳(002)晶面的晶格间距吻合,内部包裹的纳米颗粒的晶格间距为0.20 nm,与钴镍合金(111)晶面的晶格间距吻合。通过EDS mapping分析(图1e)可以看出,材料中均匀存在C、N、Co、Ni元素,说明成功实现了材料中氮元素的掺杂。

**图1:**
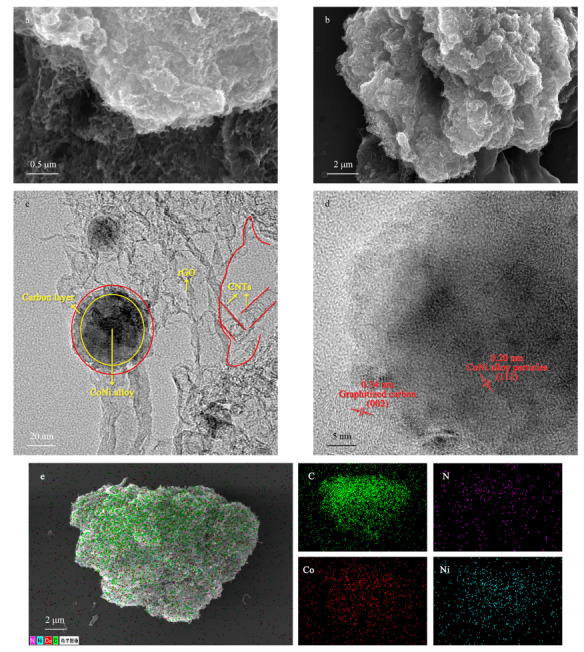
CoNi@NGC材料的(a, b)扫描电镜图、(c, d)透射电镜图和(e)能谱测试结果

通过XRD(图2a)表征发现,在26.2°出现的衍射峰对应于石墨化碳的(002)晶面,在44.4°、51.7°和77.3°出现的衍射峰对应于CoNi合金的(111)、(200)和(220)晶面,吸附前后XRD的测试结果变化不大,证明该吸附剂在吸附过程中结构稳定。通过FT-IR谱图(图2b)表征了CoNi@NGC材料在吸附BPs前后表面官能团的变化,3400~3600 cm^-1^出现O-H基团引起的吸收峰,表明该材料中含有大量的-OH,对比吸附前后O-H的吸收峰从3440 cm^-1^偏移至3438 cm^-1^,表明-OH作为氢键位点参与吸附^[22]^。2900 cm^-1^左右出现的特征峰为芳环C-H伸缩振动峰,在1590 cm^-1^附近出现芳香C=C骨架的振动带,表明石墨结构的存在,同时,由吸附后C=C的峰位移至1600 cm^-1^可看出在吸附过程中存在*π-π*共轭作用。C-N和C-O键在1270 cm^-1^左右出现的特征峰不仅能够表明样品中含有丰富的含氧官能团,还进一步证明了氮元素的成功掺杂,且C-N键从1263 cm^-1^处的伸缩振动向较大波数1270 cm^-1^有较小的位移,可以认为是氢键作用影响而致^[10,20]^。700~900 cm^-1^处出现的特征峰可以归因于C-H的弯曲振动和CH_2_亚甲基平面摇摆振动。550 cm^-1^处的特征峰可能是CoNi合金对rGO的激发作用产生的特征振动峰^[23]^。如图2c所示,通过氮气吸脱附测试得到的CoNi@NGC材料吸附前后的比表面积分别为109.42 m^2^/g和76.32 m^2^/g,平均孔体积分别为0.4942 m^3^/g和0.4136 m^3^/g,表明该吸附剂具有较大的比表面积和孔体积,有利于对污染物分子的吸附,吸附后,由于被有机物分子填充,导致其结果减小。通过VSM测试(图2d)可以看出,CoNi@NGC的饱和磁通量为17.98 emu/g,具有明显的超顺磁性,吸附后,饱和磁通量减小为14.68 emu/g,该磁力仍旧能够实现使用外部磁铁分离。

**图2:**
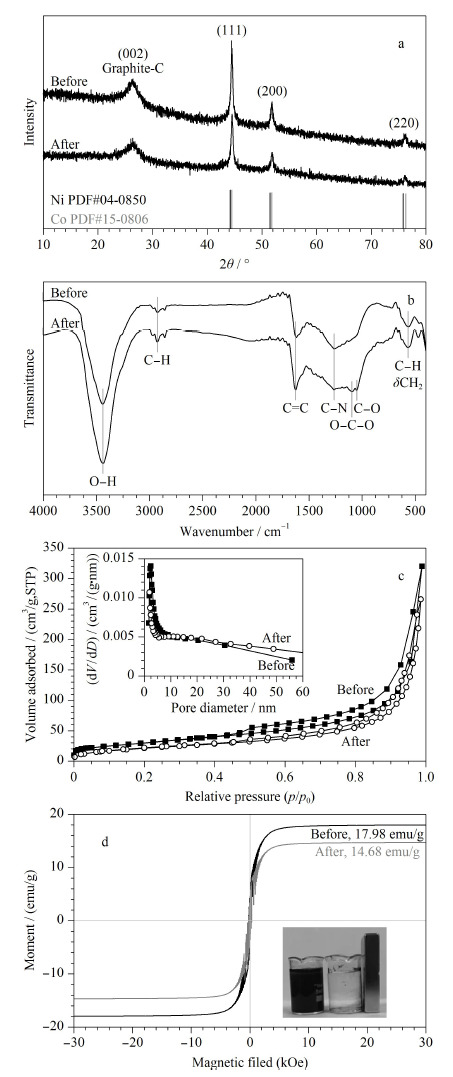
CoNi@NGC材料吸附BPs前后的(a)X射线单晶衍射图、(b)红外光谱图、(c)氮气吸脱附曲线和孔径分布曲线(内嵌)、(d)磁滞回线

通过XPS分析了吸附前后样品表面的组成和元素价态(图3a~e)。吸附前CoNi@NGC中存在Co、Ni、N、O和C元素,其中N元素的原子百分数约为7.98%(图3a)。CoNi@NGC的Co 2*p* XPS光谱(图3b)中,778.6和792.8 eV处的结合能对应于Co^0^, 781.5和795.9 eV处的结合能以及785.1和802.7 eV处的卫星峰对应于Co^2+^。Ni 2*p*的XPS光谱(图3c)中,Ni^0^的峰值位于853.2和870.8 eV, 855.1和872.2 eV处的峰值与Ni^2+^相对应,854.4、872.2 eV处的峰分别对应Ni 2*p*_3/2_和Ni 2*p*_1/2_。在吸附前后钴镍元素的峰变化不大,说明在吸附过程中,提供磁核的钴镍合金性质稳定,与XRD测试结果保持一致。在材料的C 1*s*的XPS谱图(图3d)中,284.4、285.7和288.5 eV附近的峰分别对应C-C、C-N和C-O键,N 1*s*的XPS谱图(图3e)表明,样品中含有5种形态氮:吡啶氮(398.2 eV)、钴氮(399.4 eV)、吡咯氮(400.6 eV)、石墨氮(401.2 eV)和镍氮(402.6 eV),对比吸附前后数据发现,吸附后C-C峰、吡啶氮峰、吡咯氮峰的峰面积有所增加,说明在吸附过程中吡咯氮和吡啶氮的存在有助于与污染物分子结合,有利于提供更多的*π*电子,增强材料与目标物分子间的*π-π*共轭作用,有助于提高对BPs的吸附性能^[10,24]^。

**图3:**
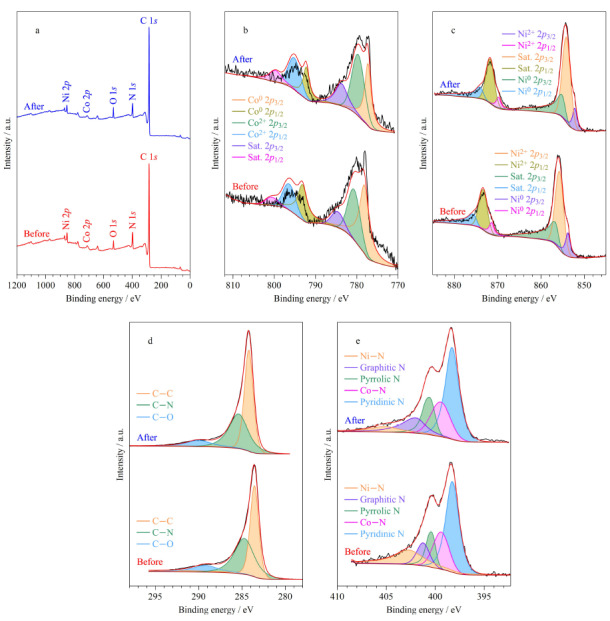
CoNi@NGC材料吸附BPs前后X射线光电子能谱分析

### 2.2 影响吸附性能的因素

#### 2.2.1 吸附剂投加量

吸附剂投加量是评估吸附剂性能的重要指标之一。为了探究投加量变化对BPs吸附性能的影响,于100 mL 5 mg/L的混合标准溶液(含6种BPs)中分别投加1、3、5、10、15 mg CoNi@NGC材料,吸附60 min,结果如图4a所示,在投加量由1 mg增至5 mg时,吸附效率逐渐增加,主要是因为随着吸附剂的增多,可吸附目标物的活性位点逐渐增多,可增加BPs的去除率。继续增加吸附剂的投加量至15 mg,去除率并未发生明显变化,其原因可能是,吸附剂含量为50 mg/L时溶液中的BPs基本吸附完全,去除率较高,因此再提高吸附剂的量去除率不会明显增加。当投加量为5 mg时,吸附60 min后BPM的去除率高达99.52%,其余5种也均在70%~97%之间。

**图4:**
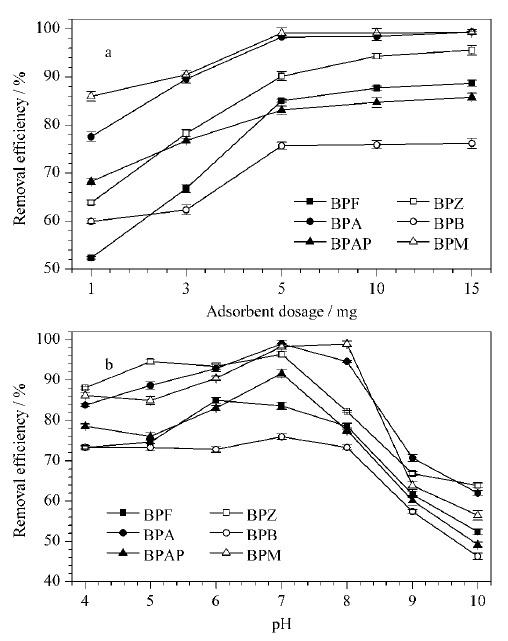
(a)吸附剂投加量和(b)溶液pH值对CoNi@NGC吸附BPs影响(*n*=3)

#### 2.2.2 pH

溶液的pH值对BPs的吸附过程具有重要影响,pH可以改变吸附剂的表面电荷和BPs在溶液中的存在形态。本实验探究了pH=4~10范围内,6种BPs去除效率的变化(图4b),当pH=7时,CoNi@NGC对6种BPs的去除率最大,超过75%,其中对BPZ、BPA和BPM的去除率超过95%。在偏酸性(pH<7)的环境下,BPs的吸附率受pH值的影响不大,在接近中性(pH为6~8)的条件下吸附效果较好,当pH>8时,吸附率明显下降。这是由于当pH>p*K*_a_时,BPs可电离为双酚盐分子,发生静电排斥作用,氢键遭到破坏,导致去除率降低。

### 2.3 吸附热力学与动力学研究

#### 2.3.1 吸附等温线

将5 mg CoNi@NGC材料分别投加到100 mL不同质量浓度(0.5、1、5、10、15 mg/L)的BPs混合溶液中,在298、308和318 K的条件下进行吸附实验,吸附60 min达到平衡后,经磁铁分离,采用HPLC-FLD测定BPs的平衡浓度,记为*C*_e_, mg/L;通过公式(1)计算所得的平衡吸附量,记为*Q*_e_, mg/g。不同温度下的吸附等温线如图5所示,CoNi@NGC对BPs的平衡吸附量*Q*_e_随着平衡浓度*C*_e_的增加而逐渐增加,随着温度的升高,*C*_e_随之减小,表明此吸附过程为放热过程。

**图5:**
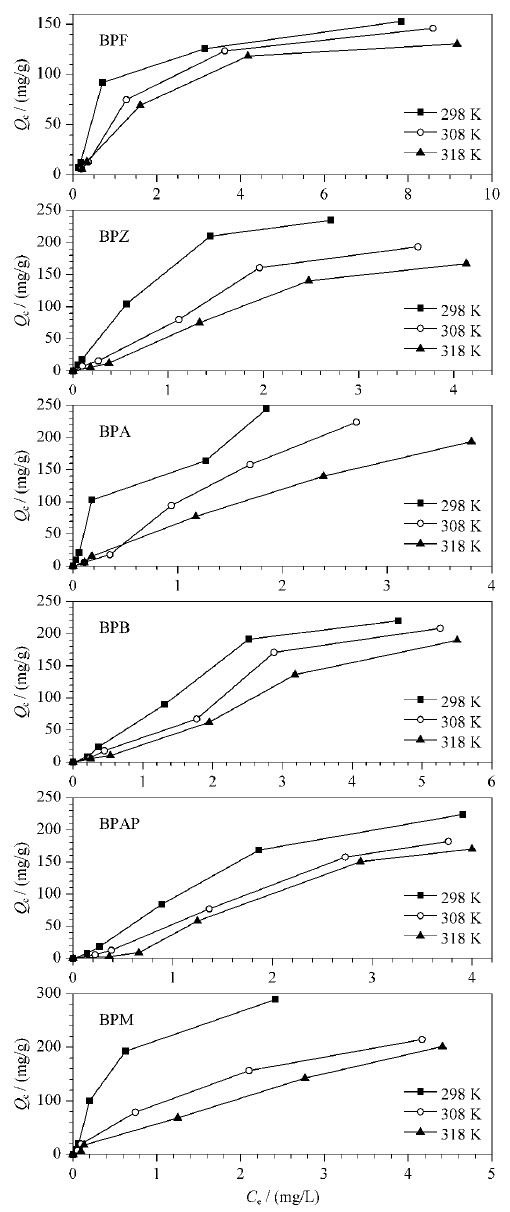
温度对CoNi@NGC吸附BPs的影响

采用Langmuir和Freundlich模型对吸附等温曲线进行拟合,结果如图6所示,Langmuir模型是假设吸附发生在单分子层,由式(3)表示;Freundlich模型是假设吸附过程是多分子层吸附,由式(4)表示:


(3) $\frac{C_{\mathrm{e}}}{Q_{\mathrm{e}}}=\frac{1}{b Q_{\mathrm{m}}}+\frac{C_{\mathrm{e}}}{Q_{\mathrm{m}}}$

(4) $\ln Q_{\mathrm{e}}=\ln K+\frac{\ln C_{\mathrm{e}}}{n}$

**图6:**
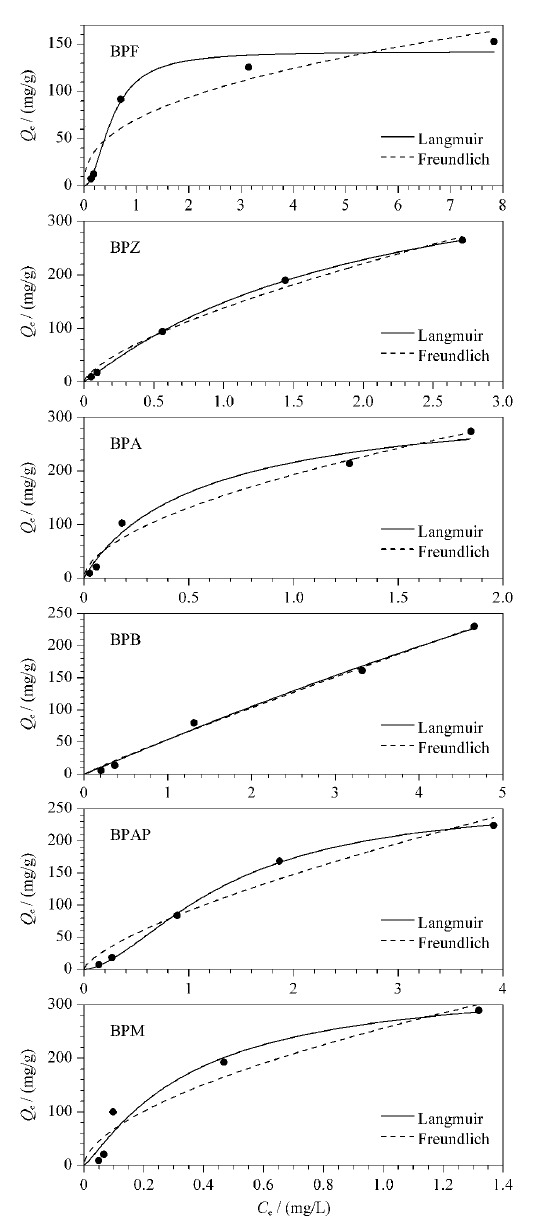
CoNi@NGC吸附等温线模型

式中,*b*为吸附平衡时的吸附常数,L/mg; *Q*_m_为理论最大吸附量,mg/g; *K*为与结合能相关的吸附常数; *n*为温度相关的特征常数。

在298 K温度条件下,通过Langmuir模型拟合可得,BPF、BPZ、BPA、BPB、BPAP、BPM的相关系数(*R*^2^)分别为0.866、0.703、0.816、0.405、0.701和0.596,通过模型计算的CoNi@NGC的理论饱和吸附量*Q*_m_依次为243.9、666.7、344.8、2000.0、769.2和588.2 mg/g。通过Freundlich模型拟合的*R*^2^分别为0.886、0.980、0.922、0.998、0.959和0.800,均大于Langmuir模型拟合结果,因此,CoNi@NGC对BPs的吸附更符合Freundlich模型。Freundlich模型中的参数*n*值反映了吸附剂的不均匀性或吸附反应的强度,当*n*接近1时,吸附等温线的非线性程度会减小。当1/*n*在0~1之间时,表明过程有利于吸附进行,新的吸附位点会随着吸附量的增加而不断生成。本实验中1/*n*均小于1(见表1),说明BPs极易吸附在材料表面,另外1/*n*范围在0.7334到0.9824之间,和BPs的性质参数之间没有明显的相关性,这可表明Freundlich模型的非线性程度是由多种因素决定的。Freundlich模型主要描述存在物理吸附和化学吸附的多分子层吸附,其中大多是非均匀的吸附。由于吸附质在吸附剂上的作用力有氢键、*π-π*共轭等,因此导致吸附的原因既有物理作用也有化学作用^[25]^。

**表1:** 298 K下CoNi@NGC吸附BPs的Langmuir、Freundlich和D-R吸附等温线参数和拟合常数

Target	Langmuir				Freundlich				D-R		
	b	Q_m_	R^2^		K	1/n	R^2^		Q_m_	E	R^2^
BPF	0.31	243.9	0.867		49.57	0.788	0.886		142.49	2.102	0.929
BPZ	0.25	666.7	0.703		128.97	0.902	0.980		255.78	2.704	0.976
BPA	1.21	344.8	0.817		182.05	0.733	0.922		222.05	3.522	0.970
BPB	0.03	2000	0.405		55.53	0.982	0.998		144.25	2.072	0.898
BPAP	0.11	769.2	0.701		71.86	0.936	0.959		158.02	2.250	0.945
BPM	0.12	588.2	0.596		316.59	0.914	0.800		360.36	3.026	0.907

*b*: absorption constant; *Q_m_*: maximum adsorption capacity, mg/g; *R*^2^: correlation coefficient; *K*: binding energy-dependent adsorption constant; *n*: temperature-dependent characteristic constant; *E*: adsorption energy, kJ/mol.

本文还使用Dubinin-Radushkevich (D-R)吸附等温线进行了研究,根据式(5)、(6)可计算出整个体系的平均吸附能(*E*):


(5) ln *Q*_e_=ln *Q*_m_-*K*ε^2^

(6) *E*=(-2*K*)^-1/2^

式中:*ε*(kJ/mol)表示吸附势能,表示一个分子移动到吸附区域所需的自由能;*K*为与吸附能相关的常数。*ε*随浓度的变化关系如式(7)所示:


(7) $\varepsilon=R T \ln \left(1+\frac{1}{C_{\mathrm{e}}}\right)$

其中,*R*为摩尔气体常数,8.314 J/(mol·K), *T*为热力学温度,K。

结果如表1所示,在298 K下求得BPF、BPZ、BPA、BPB、BPAP、BPM的平均吸附能分别为2.10、2.71、3.52、2.07、2.25和3.03 kJ/mol,均低于8 kJ/mol,因此本吸附过程主要由物理吸附主导。

#### 2.3.2 吸附热力学

采用吉布斯-亥姆霍兹公式进行吸附热力学分析,结果如表2所示:Δ*G*、Δ*H*、Δ*S*分别为吉布斯自由能(kJ/mol)、焓(kJ/mol)、熵(kJ/(mol·K))。在298、308和318 K条件下,Δ*G*均小于0,绝对值介于0~20 kJ/mol之间,Δ*H*为负值,且绝对值均小于50 kJ/mol,说明CoNi@NGC材料对BPs的吸附为自发进行的物理吸附过程,且该过程放热。Δ*S*为负值,表明材料对BPs的吸附随着吸附量的增加逐渐趋于稳定。

**表2:** CoNi@NGC吸附BPs的热力学参数

Target	Temperature/ K	ΔH/ (kJ/mol)	ΔG/ (kJ/mol)	ΔS/ (kJ/(mol·K))
BPF	298		-11.9624	-0.1011
	308	-42.0929	-10.4467	-0.1027
	318		-9.9517	-0.1011
BPZ	298		-12.7168	-0.1035
	308	-43.522	-19.9705	-0.1058
	318		-10.6635	-0.1034
BPA	298		-10.1827	-0.1084
	308	-45.5466	-9.3201	-0.1101
	318		-9.1588	-0.1084
BPB	298		-13.2377	-0.0517
	308	-25.6021	-11.6394	-0.0528
	318		-11.0809	-0.0511
BPAP	298		-10.1828	-0.1252
	308	-48.414	-9.3201	-0.123
	318		-9.1589	-0.1252
BPM	298		-11.1121	-0.0136
	308	-14.7964	-10.5147	-0.013
	318		-8.5938	-0.0136

#### 2.3.3 吸附动力学

如图7所示,随着时间的延长,6种BPs去除率的变化均呈现先快后慢的趋势。其中,对于BPA和BPM而言,在0~5 min吸附快速且高效,在5 min时达到吸附平衡且去除率分别高达98.21%和99.01%;在90 min时,对二者的去除率接近100%。对于BPF、BPZ、BPB和BPAP而言,在0~10 min去除率迅速增加,随后吸附位点逐渐饱和导致吸附缓慢,在90 min时除BPB的去除率为73.94%外,其余3种BPs的去除率均高于80%。

**图7:**
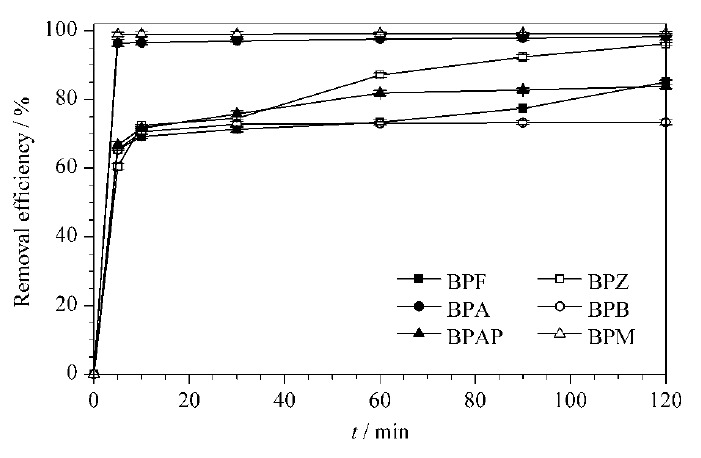
去除率随时间的变化曲线(*n*=3)

为进一步研究吸附过程,采用准一级动力学模型和准二级动力学模型分析吸附BPs的动力学数据,结果如图8a,b和表3所示,准二级动力学拟合所得*R*^2^大于0.995,均大于准一级动力学模型拟合所得的*R*^2^,说明准二级动力学模型可以更好地描述吸附过程,这也说明CoNi@NGC对BPs吸附的速率控制步骤以化学吸附为主。用准二级动力学速率常数*k*和吸附量*Q*计算初始吸附速率*h* (mg/(g·min)),计算公式如式(8):


(8) *h*=*k*^2^*Q*^2^

**图8:**
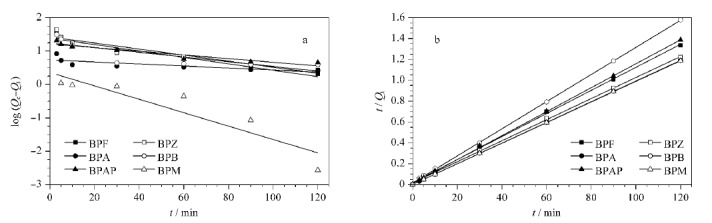
(a)准一级动力学模型图和(b)准二级动力学模型图

**表3:** 298 K下CoNi@NGC吸附BPs的动力学参数

Target	Experiment Q_e,exp_/(mg/g)	Pseudo-first-order kinetic				Pseudo-second-order kinetic			
		k/min^-1^	Q/(mg/g)	R^2^		k/((mg/g)^-1^·min^-1^)	Q/(mg/g)	R^2^	h/(mg/(g·min))
BPF	65.54	0.025	91.95	0.932		0.005	90.91	0.999	0.21
BPZ	59.29	0.026	101.06	0.952		0.033	100	0.995	10.89
BPA	41.09	0.004	103.48	0.987		0.07	101.01	0.999	49.99
BPB	23.31	0.02	79.92	0.665		0.013	76.92	0.999	0.99
BPAP	44.03	0.018	91.14	0.714		0.01	86.96	0.999	0.76
BPM	2.51	0.04	101.05	0.853		0.089	101.01	0.999	80.82

*Q*_e,exp_: experiment equilibrium adsorption quantity; *k*: adsorption rate constant; *h*: initial adsorption rate.

从表3可知,BPZ、BPA和BPM的初始吸附速率*h*分别为10.89、49.99和80.82 mg/(g·min),表明CoNi@NGC对这3个污染物的吸附过程较快且有着高密度的活性吸附位点。

#### 2.3.4 吸附速率控制机制

吸附过程中存在外部扩散和粒子内扩散两个阶段,其中较慢的一个为吸附速率的主要控制步骤。用Weber-Morris模型来研究扩散在吸附过程中的作用,表达式如式(9, 10):


(9) *Q*_t_=*K*_d_*t*^1/2^+*I*

(10) *K*_d_=*Q*_e_/*C*_e_

式中*K*_d_表示粒子内部扩散速度常数(mg/(min^0.5^·g)), *K*_d_值越大,表明吸附质在吸附剂内越容易扩散。*I*值表示与边界层厚度相关的常数,*I*值越大,则边界层效应越明显。用Weber-Morris模型对吸附进行分线段拟合(参数见表4),如图9所示,结果表明吸附过程主要分为3个阶段:第1阶段为外部扩散阶段,BPs固定在CoNi@NGC表面的吸附位点上;第2阶段为粒子内扩散阶段,BPs被吸附在CoNi@NGC的孔隙和微孔之中;第3阶段通常是一个吸附-解吸平衡的过程。由于扩散模型所拟合的直线并未经过原点,因此CoNi@NGC吸附BPs的机理相对而言比较复杂,粒子内扩散和外部扩散共同影响着反应速率。

**表4:** Weber-Morris模型分段拟合参数

Target		External diffusion								Internal diffusion								Adsorption equilibrium				
		K_d_		I		R^2^				K_d_		I		R^2^				K_d_	I		R^2^	
BPF	14.392		33.542		0.938				1.835		71.638		0.999				1.264		76.393		0.937	
BPZ	17.499		30.241		0.869				2.35		77.172		0.904				1.273		84.65		0.978	
BPA	2.889		90.767		0.856				0.425		98.88		0.937				0.394		99.621		0.777	
BPB	10.876		31.117		0.956				0.333		74.943		0.947				0.172		75.479		0.774	
BPAP	4.535		63.639		0.897				5.533		68.042		0.999				0.343		82.917		0.918	
BPM	0.065		99.871		0.999				0.056		100.24		0.997				0.004		100.28		0.999	

*K*_d_: rate constants of Weber-Morris model, mg/(min^0.5^·g); *I*: the constants describing the effects of the boundary layer.

**图9:**
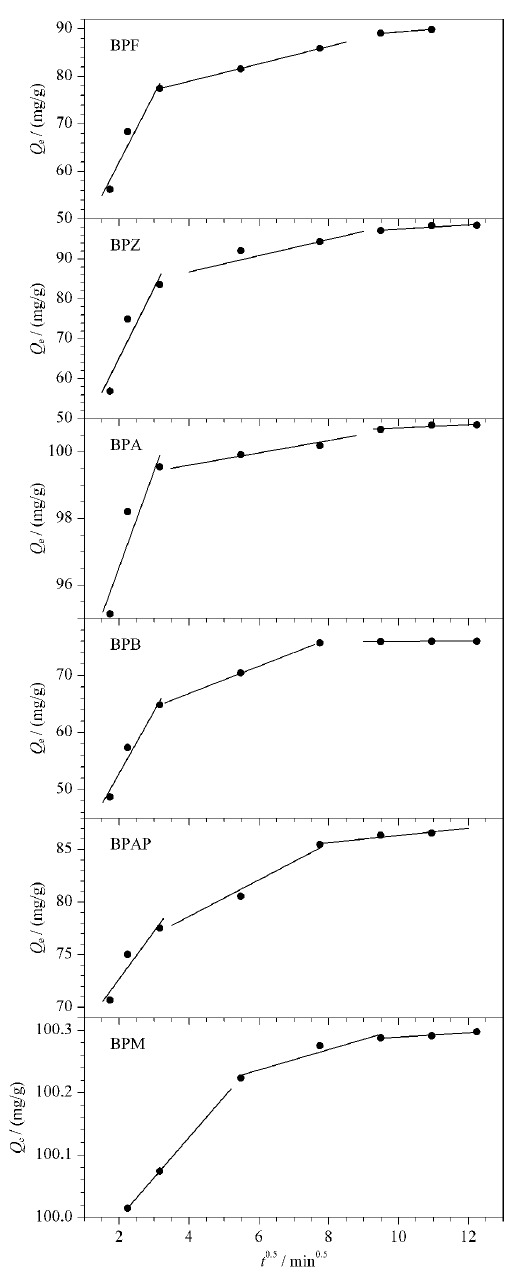
Weber-Morris模型分段拟合曲线

#### 2.3.5 再生性能研究

吸附材料的再生性能在实际应用中十分关键。本实验将吸附后的材料用磁铁分离,用乙醇和超纯水交替洗涤3次,60 ℃下烘干,将烘干后的CoNi@NGC继续在最优条件下用于吸附实验。结果如图10所示,经过5次吸附脱附后,对6种BPs的去除率的损失范围为3.14%~12.35%,这表明所开发的CoNi@NGC是一种循环使用性好、吸附效率高且便于回收利用的优异吸附剂。

**图10:**
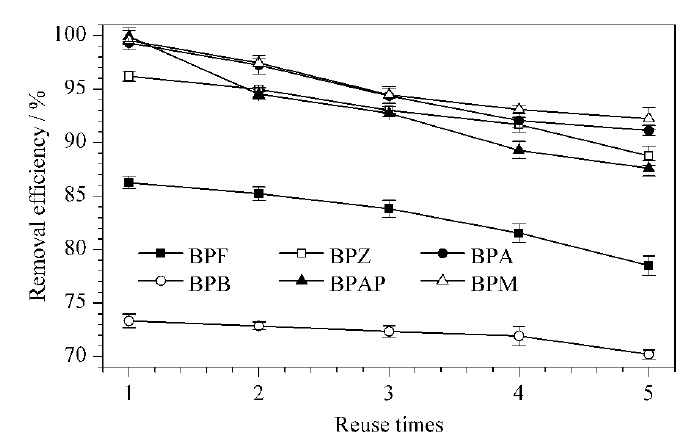
吸附剂的吸附再生性能(*n*=3)

#### 2.3.6 吸附机理分析

吸附实验结果表明,CoNi@NGC材料对BPs的吸附过程中物理吸附占主导,这主要得益于石墨烯和碳管互相结合构建的三维结构增大了材料的比表面积,丰富了孔结构,有利于BPs分子快速与材料表面的活性吸附位点接触。通过吸附前后的FT-IR和XPS比较发现,材料的结构中富含含氧官能团且在材料中成功掺杂有吡咯氮、吡啶氮,这些有助于增强材料与污染物分子间的*π-π*共轭作用,可促进吸附剂材料吸附BPs分子,并在较短时间内达到平衡,大大提高吸附效率。吸附的整个过程是范德华力、*π-π*共轭、氢键等共同作用的结果。优异的三维结构和丰富的活性位点大大提高了材料的吸附容量,以双酚A为例,相较于前期报道的Ni-NMCNTS吸附剂的饱和吸附容量73.26 mg/g^[10]^, CoNi@NGC的饱和吸附容量约为其1.4倍,磁性也较强。

### 2.4 泡腾辅助分散固相微萃取结合HPLC-FLD检测方法的构建

#### 2.4.1 萃取条件优化

基于材料对BPs吸附过程的探究结果,选择投加量为5 mg、pH=7条件进行萃取因子的优化。为验证泡腾反应对萃取回收率的影响,在相同的实验条件下比较了制成泡腾片剂与直接加入萃取剂的情况下相同时间6种BPs的平均萃取回收率(ERs)。如图11a所示,在不存在泡腾反应的情况下,6种BPs的平均ERs明显低于存在泡腾反应的情况。因此,可以推断出泡腾反应有助于短时间内将纳米材料很好地分散,从而在短时间内增加萃取剂与目标物的接触面积,对提高ERs有很大的帮助。CoNi@NGC吸附BPs后,使用外部磁铁将吸附有BPs的材料收集至试管底部,弃去上清液,将收集得到的材料选用合适的洗脱剂进行洗脱。选择甲醇、乙醇、丙酮和乙腈4种洗脱剂进行比较,结果如图11b所示,平均ERs为丙酮>乙醇>乙腈>甲醇,此外丙酮的极性为5.4,丙酮的极性与中等极性的双酚类化合物相似^[20,21]^。因此,使用丙酮作为洗脱剂。

**图11:**
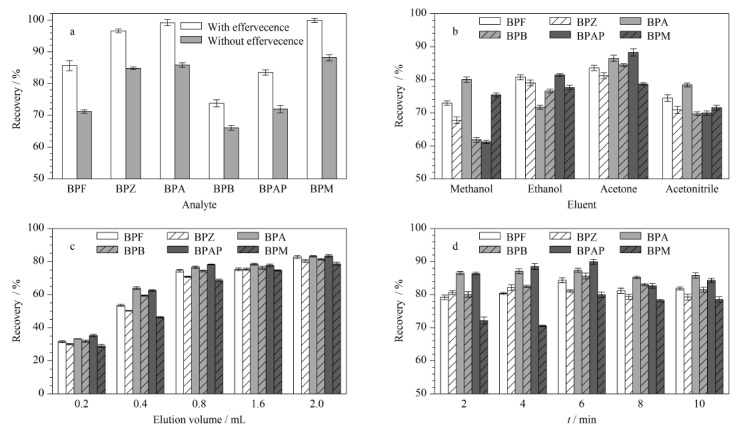
(a)有无泡腾片剂、(b)洗脱剂种类、(c)洗脱剂体积和(d)洗脱时间对BPs萃取性能的影响(*n*=3)

对洗脱剂的体积进行了优化,选择洗脱体积为0.2~2.0 mL进行研究,结果如图11c所示,平均ERs随着洗脱剂体积的增加而增加,当洗脱体积为2.0 mL时,6种BPs的平均ERs达到81.67%。因此,实验选择2.0 mL丙酮对吸附后的材料进行洗脱。在2~10 min的范围内对洗脱时间进行了优化(图11d),当洗脱时间从2 min增至6 min时,随着洗脱时间的增加,平均ERs也逐渐增加,当时间超过6 min时,平均ERs却逐渐降低,这可能是由于当洗脱时间到达6 min时,达到了BPs吸附-解吸平衡,过多的洗脱时间可能导致BPs在溶液中有更多的解吸和溶解,从而使得ERs下降。

#### 2.4.2 方法评价

在优化的条件下(吸附剂投加量5 mg, 2 mL丙酮洗脱6 min, pH=7),通过线性关系、检出限(LOD, *S/N*=3)、定量限(LOQ, *S/N*=10)以及日内、日间精密度(RSD)对实验方法进行了评估。如表5所示,6种BPs的*R*^2^值为0.9936~0.9993,在0.33~500 μg/L范围内具有良好的线性关系。6种BPs的LOD和LOQ分别为0.06~0.20 μg/L和0.20~0.66 μg/L。日内和日间精密度分别为1.44%~4.76%和1.69%~5.36%。

**表5:** CoNi@NGC基泡腾辅助分散固相微萃取结合HPLC-FLD方法对BPs的检测性能

Analyte	Linear range/ (μg/L)	Regression equation/(μg/L)	R^2^	LOD/ (μg/L)	LOQ/ (μg/L)	Intra-day RSDs/%(n=6)				Inter-day RSDs/%(n=6)		
						Low	Medium	High		Low	Medium	High
BPF	0.66-500	y=3.34×10^3^x-2.64×10^2^	0.9993	0.11	0.36	3.41	1.37	1.52		4.78	4.43	3.91
BPZ	0.60-500	y=3.04×10^3^x+4.95×10^4^	0.9977	0.18	0.60	1.76	4.33	3.97		3.46	2.94	2.27
BPA	0.66-500	y=3.32×10^3^x+2.91×10^4^	0.9966	0.20	0.66	2.57	4.76	2.79		3.53	2.79	1.98
BPB	0.63-500	y=2.84×10^3^x+5.93×10^4^	0.9966	0.18	0.60	3.81	2.08	3.16		5.36	4.99	3.16
BPAP	1.00-500	y=1.69×10^3^x+7.05×10^4^	0.9936	0.10	0.33	2.56	1.44	4.02		2.68	2.07	2.53
BPM	0.33-500	y=7.59×10^3^x+5.57×10^5^	0.9950	0.06	0.20	2.04	3.39	1.99		1.69	3.85	2.91

*y*: peak area; *x*: mass concentration, μg/L; LOD: limit of detection at *S/N*=3; LOQ: limit of quantification at *S/N*=10; The detailed experimental conditions were as follows: (1) BP standards were fortified in peach juice as an example; (2) extraction process: 150 mg of tartaric acid (TTA) and 106 mg Na_2_CO_3_ as precursors of an effervescent tablet, 5 mg of CoNi@NGC as extractant, pH 7, 2.0 mL of acetone as eluent solution and 6 min elution time.

#### 2.4.3 实际样品分析

为了探究所建立方法的实际应用性,将该方法用于检测盒装饮料中的痕量BPs,在桃汁中检测到BPA和BPB分别为2.09 μg/L和1.37 μg/L,其余均未检出。在20 μg/L的添加水平下,6个盒装饮料样品的色谱图如图12所示,这表明,在盒装饮料中BPF、BPZ、BPA、BPB、BPAP和BPM均分离很好。所有盒装饮料样品中,在5、20、200 μg/L加标水平下的回收率为82.4%~103.7%(见表6),这些结果表明,新开发的方法能够以高精密度和准确度满足实际盒装饮料样品中痕量BPs的检测要求。

**图12:**
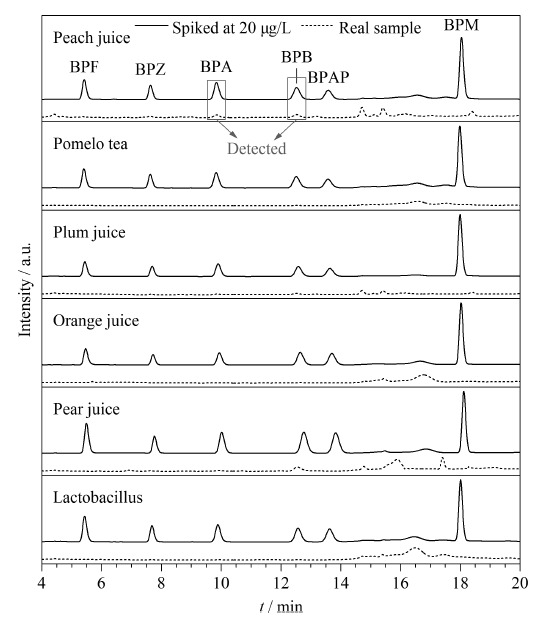
实际样品和加标样品的色谱图

**表6:** 6种BPs在果汁样品中的加标回收率(*n*=3)

Sample	Added/ (μg/L)	Recoveries/%					
		BPF	BPZ	BPA	BPB	BPAP	BPM
Peach juice^*^	5	94.6±2.1	93.4±3.8	86.2±4.3	92.3±2.7	90.0±1.9	83.7±3.2
	20	87.2±4.3	94.7±2.2	88.7±3.5	98.±3.1	90.2±4.2	88.5±3.5
	200	92.3±3.7	96.5±2.9	95.2±2.7	86.3±2.0	85.9±4.0	86.4±2.7
Pomelo tea	5	84.8±2.5	89.1±3.3	92.4±4.4	92.0±3.6	87.7±3.2	87.9±1.9
	20	103.1±4.7	87.5±4.6	93.3±2.9	87.8±2.5	88.9±1.7	86.1±2.4
	200	93.5±2.6	105.3±3.1	90.6±1.5	85.3±2.2	94.3±3.4	90.4±4.5
Plum juice	5	98.1±3.4	97.3±2.4	87.1±2.8	86.5±3.9	96.9±1.8	91.3±4.8
	20	90.6±4.2	94.7±3.8	85.9±3.1	91.8±3.7	82.4±2.6	84.4±2.8
	200	91.7±2.4	84.2±2.9	99.2±4.2	101.2±2.0	92.3±4.6	96.0±3.7
Orange juice	5	85.8±1.7	82.7±1.5	87.4±2.9	94.5±2.1	92.6±3.9	90.4±3.4
	20	92.4±3.9	99.3±4.8	85.1±1.0	87.3±4.5	88.9±4.1	99.6±2.6
	200	101.3±1.9	96.4±4.1	92.1±4.2	85.7±2.9	83.2±1.5	103.7±2.1
Pear juice	5	91.2±2.2	88.1±2.7	100.5±3.7	94.1±4.0	96.7±3.6	85.0±4.0
	20	83.9±4.3	87.5±4.2	93.5±2.6	90.9±3.9	88.0±2.5	93.7±2.8
	200	99.2±1.4	93.3±3.6	94.8±1.7	98.2±2.1	87.3±1.4	96.1±1.6
Lactobacilus	5	85.3±2.0	89.0±1.2	94.6±2.9	96.5±4.4	94.8±3.9	88.2±2.5
	20	88.4±1.2	90.8±2.5	93.9±3.1	98.1±2.8	93.1±3.6	85.2±3.6
	200	93.2±3.1	93.1±3.3	102.4±3.0	97.3±3.4	95.1±2.2	94.1±3.0

* Background: BPA 2.09 μg/L, BPB 1.37 μg/L.

与已报道的同类方法相对比,该方法有以下优点:(1)能够同时检测6种污染物,相较其他方法污染物种类更多^[26⇓-28]^; (2)萃取时间仅需要6 min,而其他方法需要10~20 min^[26,27]^; (3)该方法的检出限和回收率可与MSPE-超高效液相色谱-MS方法^[26]^相媲美,但是操作更加简单,预处理成本也更低。总之,该方法在饮料等复杂基质的实际样品中具有较大的应用潜力。

## 3 结论

本研究以简单煅烧法合成了包裹钴镍合金的氮掺杂石墨烯与碳纳米管复合材料,该材料比表面积大、孔径丰富,具有较多的吸附活性位点。将该材料用作吸附剂吸附水中的双酚类污染物,结果证明该吸附过程符合Freundlich模型和准二级动力学模型,主要涉及范德华力、*π-π*共轭和氢键的共同作用。同时该吸附剂具有良好的可再生性能,饱和磁化力高,使用过程中便于分离,具有良好的实际应用价值。将该材料作为萃取介质与泡腾片剂辅助磁性固相微萃取技术相结合用于6种盒装饮料中BPs检测,具有灵敏度高、萃取速度快、环境友好的等优点,具有较好的应用前景。
